# Examining illness narratives in the context of the postoperative psychological state: A mixed-methods study of emotion-focused illness narrative

**DOI:** 10.1186/s13030-024-00318-4

**Published:** 2024-10-12

**Authors:** Tünde Lévai, György Lázár, Erna Krajinovic, Iván Devosa, Melinda Látos

**Affiliations:** 1 Department of Surgery, Albert Szent-Györgyi Health Centre, University of Szeged, Szeged, Hungary; 2 Institute of Pedagogy, Department of Art, Physical Education and Lifestyle, Károli Gáspár University of the Reformed Church, Budapest, Hungary; 3 Institute of Psychology, Department of Personality, Clinical and Health Psychology, University of Szeged, Szeged, Hungary

## Background

### Chronic illness as a traumatic experience

The examination and therapeutic approaches of illness narratives have become increasingly prominent in recent decades [11, 19, 25, 41]. Trauma caused by somatic, chronic illness can damage the integrity of the body and the self simultaneously, requiring the sufferer to adapt and develop a new relationship with their body, self and social environment [2, 19, 26, 33]. Through chronic illness, the continuity of life is disrupted, the narrative coherence of the life story is broken, and this traumatic structured crisis can result in a loss of emotional balance and a negative shift in emotion regulation. This makes it difficult to adaptively cope with the psychological distress and physical symptoms of illness in both the short and long term [9, 23, 33, 41].

Chronic disease patients and cancer patients share traumatic experiences, but the specific experiences of each disease have their own characteristics [41, 43]. The important psychological consequences of cancer are that patients may develop a strong fear of death and a strong fear of recurrence, fears that are often based on physical suffering, depending on the type of cancer [56]. Vehling and colleagues [56] found that, in a sample of patients with advanced cancer, death-related anxiety was very common, with 22–55% of their patients having at least moderate levels of anxiety. Medical interventions such as surgery and oncological treatments, which are part of the recovery from cancer, may also lead to further traumatization due to their side effects and negative impact on body image [15, 43].

### Illness narratives theory

Narrative theory provides an innovative and beneficial way to study the subjective experience of illness [1, 29, 19]. According to the guiding theory of Arthur W. Frank, illness narratives allow us to learn about the subjective meanings constructed around illness [18–20]. Based on Frank’s definition, three illness narratives can be distinguished [18–20].

These narratives are fragmented stories that do not fit into the structural framework of a story; emotions break the narrative, and the stories are about the disintegration of the previous self-image and structures of meaning [19, 20]. The chaos narrative portrays the person as a passive victim, revealing vulnerability and futility [19]. The illness experience is characterised as empty, meaningless and devoid of purpose.

The restitution story is about illness, suffering, and treatment: health is restored through therapy, and healing is achieved [19, 20]. This narrative reflects a modernist expectation that there is a remedy for every suffering and that the illness is an unfortunate breakdown in the body [57]. The restitution narrative’s basic plot is that “I was healthy yesterday, and now I am sick, but tomorrow I will be better” [19, 20].

The quest story, in which illness is experienced as a kind of mission or research, is a condition in which something can be learned, something can be discovered, and this knowledge can be passed on to others [19, 20]. Quest narratives are heroic; they involve perseverance and are oriented to the future. In quest narratives, events are intertwined, and illness can become a motivator for social action or can be expanded to reveal fate [19, 20].

Whitehead [57] examined the narratives of patients with chronic fatigue syndrome and myalgic encephalomyelitis (CFS/ME) and found that patients portrayed the chaos narrative as expressions of depression, anger, and isolation. The prolonged dominance of this narrative was presumably explained by the uncertainty of the patients’ prognosis and the uncertainty of effectiveness of their treatments, with patients not believing they would be cured, in contrast to other patient groups such as HIV or breast cancer. People with CFS/ME do not as actively embrace the restitution narrative, and it can be speculated that this is related to the constant reminder of the illness that they live with; the body is never asymptomatic [57]. Nettleton and colleagues [36], examining the narratives of patients with medically unexplained symptoms, also found a dominance of the chaos narrative, which was based on the assumption of a lack of accurate medical diagnosis, whereby patients could not adequately interpret and thus reconstruct their condition, their physical symptoms having no origin in the absence of diagnosis.

Schoenau [45] investigated the narratives of operable lung cancer patients through interviews, and she showed that ‘being lucky’ was the dominant narrative about operable lung cancer, based on the patients’ perceived lack of control over the disease, passive patient role, and active medical role, an overarching narrative theme that could be considered a restitution illness narrative in Frank’s interpretation, due to its focus on recovery. Thomas MacLean [51] examined the illness narratives of breast cancer patients, according to Frank’s categorisation and found the restitutiton narrative to be the most desirable narrative, followed by the chaos narrative, with the quest narrative being the least frequent for patients. Ratcliff and colleagues [41] investigated the recovery pathways of patients healing from different cancer types using qualitative and quantitative methods simultaneously, with patients in the ‘Never the same path’ narrative group having the highest levels of anxiety and depression and the lowest levels of post-traumatic growth compared to the other groups.

In an editorial, Soundy [48] draws attention to the need to understand emotions, hope, and psychological adaptation within narratives about illness: mapping the master plots of the individual and understanding their psycho-emotional content can provide an essential tool for understanding the mental well-being of patients. Further research is needed to explore and consider these points further [36, 41, 48]. However, as we have seen from the research results presented above, the current literature mainly examines and categorizes illness narratives through thematic narrative theories, focusing on the structure of the narrative and meaning of the disease, and there is a lack of literature that uses complex, e.g. mixed methodologies, to investigate the psychological characteristics underlying patients’ illness narratives [21, 51, 45, 57]. Frank’s conceptualisation provides an understanding of illness narratives that can be used to make sense of the development of the psychological factors that accompany the illness process, This was an essential theoretical framework for our present research in which we used quantitative measures to explore the psychological factors that characterise the postoperative state in an attempt to address a gap in the literature [45, 48, 57].

### The use of qualitative methods to study illness narratives: The advantage of timeline-based techniques

Through qualitative research, we can effectively capture the complex, personal phenomenology of the experience of illness described above because these investigations facilitate memory processes and the communication of difficult bodily experiences and emotions to articulate by allowing us to go beyond the limits of verbality. Buckley et al., [10, 37, 47]. Qualitative methods can open the way to elusive experiences such as becoming ill, existential and psychological loss, and damage to the integrity of the body and the self [13, 30, 31]. A multifaceted understanding of the subjective experience of somatic illness and its physical and psychological characteristics can be facilitated by a narrative psychology approach, where narrative can become a tool for the study of complex psychological processes [8, 11, 12, 52, 51, 39].

Graphic, visual elicitation techniques can capture and sometimes evoke untold content, thus creating narratives. When using these techniques, the subject is asked to create a visual artwork, usually a picture or drawing [31, 38, 52]. Drawing autobiographical timelines chronologically plotting significant events helps restructure an individual’s life story, which is particularly important when coping with a chronic disease [31, 47, 52]. Visual elicitation techniques can transcend the boundaries of verbality to access the experiences of a person suffering from an illness and effectively facilitate the verbalisation of specific sensitive topics, such as painful physical experiences and distressing emotions [31, 47, 48, 52]. There are several examples of the use of graphic visual elicitation techniques to explore disease narratives, such as [31] who investigated the meaning-making characteristics of cancer patients using an autobigoraphic timeline and Thygesen and colleagues [52] who investigated changes in the emotional state of patients with gynaecological tumour during their disease process by constructing a graph. Except for the illness narratives defined by Frank, to the best of our knowledge there are currently no studies in the literature that use a graphic visual elicitation technique to map the content of the three narrative types [31, 52].

### Objective

The present study investigates illness narratives and their emotional aspects regarding their association with postoperative psychological status. Research in recent years has produced a growing body of knowledge on the content and structures of the three types of illness narratives identified by Frank and which features of illness within particular patient groups; availability of effective therapy, certainty of prognosis, experience of being out of control; determine the dominant illness narrative [36, 45, 51, 57]. However, what is currently unknown in the literature is the psychological state of chaos, restitution, and the quest narrative in terms of depression, anxiety, perceived stress, illness perception, and quality of life among patients who have undergone surgery.

Our objective was to examine the associations of emotion-focused illness narratives with postoperative emotional and mood state, perceived stress, quality of life and illness perception. Our primary research question was: What are the differences between chaos story, restitution story, and quest story groups regarding postoperative psychological status? Based on the available and previously presented literature, we hypothesized that (1) patients who use chaos narrative would be characterized by the most negative psychological status and that they would be the most likely to show a great psychological burden as a result of their illness. We further hypothesized that (2) patients using the restitution narrative would be characterized by a moderate psychological state compared to the other two narrative types, with these patients showing less psychological distress than those using the chaos narrative. Finally, we hypothesize that (3) patients using the quest narrative would be characterised by the most positive psychological state, with the least psychological burden caused by their disease compared to the other two narrative types.

## Methods

### Study design

Our research was based on an observational, cross-sectional study design, following a qualitatively-driven mixed-methods design that simultaneously used qualitative and quantitative measurement tools. The complete study sample was made up of patients who had undergone surgery, with each subject tested once, within 5 days of surgery. The test session included completing a questionnaire package that assessed the postoperative psychological status, followed by a semi-structured interview that used the Emotional Graph of Illness Trajectory. This graphical elicitation technique is used to explore the emotion-focused illness narrative.

### Data collection procedure

The test enrollment period of our study ran for 20 months, from September 2020 to May 2022. The single, face-to-face test session took place in our surgical clinic within five days after surgery, adapting to the constraints of the length of hospital stay and considering the patient’s physical condition.

Participation in the study was voluntary, and the study was conducted with the patient’s written informed consent. The criteria for inclusion in the study sample were: over 18 years of age, a diagnosis of chronic illness or malignant tumour, surgery in the last five days for a chronic disease or cancer, and literacy skills sufficient to read and write. Exclusion factors included severe psychiatric and neurological illness. The research was carried out following the Declaration of Helsinki and with the approval of Regional Research Ethics Committee (RKEB) of the University of Szeged, Albert Szent-Györgyi Health Centre, approval number 145/2020-SZTE (World Medical Association, Helsinki, 1964).

### Participants

Our study included 140 patients; 87 women (62.14%) and 53 men (37.86%), mean age 53.1 years (SD:12.03); who had undergone surgery and had a chronic disease or malignancy. We used access-based sampling. Their sociodemographic data showed marital status to be distributed as follows: 9.3% were single, 25% were an unmaried couple, 52.1% were married and lived with their spouse, 1.4% were married but not living with their spouse, 5.7% were divorced, 5% were widowed, and 0.7% indicated another marital status. For educational attainment: 11.5% had level eight primary qualifications, 61.1% had intermediate qualifications, and 27.4% had tertiary qualifications. Of the participants, 1.4% lived in the capital, 70% in a town, and 28.6% in a village. Regarding employment status, 32.4% were active workers, 22.3% were on sick leave, 13.7% were on a disability pension, 3.6% were unemployed, 23% were retired, and 5% were other.

The subjects had been diagnosed with their disease for 2.62 years on average (SD: 5.05) and were classified into five main groups according to their diagnosis (Table 1). In our study, the primary criterion for the design of the study sample was the postoperative status, which for all patients was a successful elective postoperative status: our study sample did not include inoperable patients. It is a characteristic of all surgery that it is a burdensome experience for the patient that can bring permanent changes to his/her life, but the positive impact of successful surgery on recovery and physical health can be attributed to its positive psychological meaning, depending on the patient’s perception [24, 40]. Although chronic disease and cancer have different courses, we combined our subjects with cancer and chronic disease into one group based on the assumptions described above. On the same basis, we did not further differentiate patients with cancer according to the stage of the disease: in general, the operability of a tumor and successful removal implies that the disease is not advanced, the stage of the disease makes the patient suitable for curative treatment, and the patient is not in the palliative phase [46].

**Table 1:** Description of the study sample by patient group

Patient group by diagnosis	*N*	Type of surgery (*N*)	Sex (*N*)	Mean age (SD)
Breast cancer	41	Mastectomy (16) Excision (25)	female (41) male (0)	53.5 (8.9)
Gastrointestinal tract cancer	36	Resection (27) Gastrectomy (2) Resection with stoma creation (7)	female (18) male (18)	56.7 (11.72)
Gasztrointestinal tract disease	21	Resection (10) Appendectomy (7) Stoma closure (2) Resection with stoma creation (2)	female (9) male (12)	40.52 (12.0)
Vascular disease	25	Stent placement (13) Amputation (4) Thrombendarterectomy (4) Bypass operation (4)	female (12) male (13)	56.12 (11.31)
Lung cancer or chronic lung disease	17	Lobectomy (10) Resection (7)	female (9) male (8)	54.41 (12.45)

### Psychological measurement tools

#### Sociodemographic data

Within the test battery used, a block of sociodemographic questions was designed to assess the age, sex, employment status, place of residence, education, and marital status of the respondents.

#### Medical data

We recorded the type of surgical intervention and data on the somatic disease that justified the surgical intervention (diagnosis, onset of disease).

#### Emotional Graph of Illness Trajectory

This graphical elicitation technique was used to identify the type of emotion-focused illness narrative used and to explore the content of the narrative. The technique can discover the dominant emotion characterising the disease process and the emotional impact of disease-related events [52]. The technique used by our research team was based on the graphical procedure of [52] a graphical elicitation technique with a timeline structure that uses a grid-graphic tool, including a horizontal axis representing events in chronological order and a vertical axis with a 0-100% coverage to assess the intensity of the emotion experienced. The national validation article of the Emotional Graph of Illness Trajectory that our research group has prepared is under publication.

During the test, the patient was first asked to name the most potent emotion they had experienced during the illness. In the next step, the patient was asked to list, in chronological order along the horizontal axis, emotionally significant events they experienced in the course of the disease and then to rate, on a scale of 0-100%, the strength of the emotion experienced during these events along the vertical axis. It was up to the patient to construct the graph during the test session. While the patient was making the graph and sharing information about it, the investigator took notes of this information on a marking sheet developed by our research team. After the patient had named all the events he/she wished to indicate on the horizontal axis and rated the intensity value, as a percentage, of the emotion associated with the events, the investigator calculated and recorded on the marking sheet the average value of the emotional intensity from the emotion intensity scores given by the subject. In this way, the quantification of the Emotional Graph of the Illness Trajectory was obtained as the average value of the emotional intensity of the graph.

Although our research was based on the original procedure and test-taking sheet of the elicitation technique created by [52], we made some modifications and innovations to enhance our tool: (1) the horizontal axis of our instrument does not contain predefined standard events characteristic of the disease process, (2) the patient determines the onset of the disease process, and (3) the grid covers the entire area between the two axes in the form of equally sized squares. The grid area given to the patient was A/4 sheet size, covering the entire sheet area, with the instructions included. In addition, we created a marking sheet on which the investigator records information related to the emotion selected by the patient (reason for the emotion, name of the associated events, description of the content and the intensity of the emotion experienced during the event). The changes made to the instrument were motivated by our research team’s objective of using it to better understand the patient’s subjective assessment of the onset of illness and to better understand the patient’s self-constructed illness narrative that was structured around self-reported events that they considered significant, so we did not include formal events on the horizontal axis of the graph. A grid area covering the entire area between the axes was included to help plot the graph and to mark the numerical values more accurately.

**Fig. 1:**
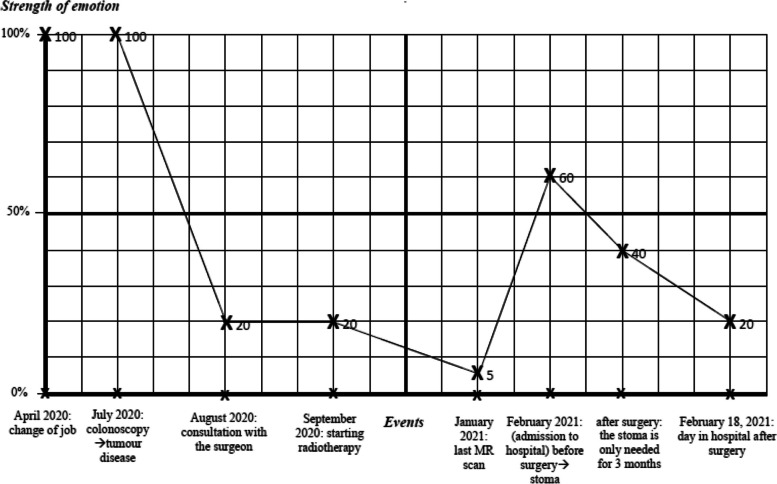
The Emotional Graph of Illness Trajectory of patient A. *Note*: A was a 40 year old female patient in a surgical inpatient ward who did the graph within five days after the resection of a malignant rectal tumour. The patient's graph depicted the evolution of a feeling of hopelessness, with an average intensity value of 45.63% during the period of the disease that ended days after the surgical procedure

The Emotional Graph of Illness Trajectory was further supplemented with a post-test based on the interview questions of the original method [52]. The questions were designed to find out what other individual or socio-environmental factors, beyond the impact of the events related to the illness experienced and described, might have played a role in the change in the intensity of emotion. The post-test questions are “*What could have caused the rise/fall of the graph?“; “If there were turning points*,* what caused them?“; “What did the patient do to change the low/high points?*“. The investigator also recorded the answers in the space provided on the marking sheet (Fig. 1).

#### State-Trait Anxiety Inventory

Used to measure anxiety levels, we applied the Spielberger Trait and State Anxiety Scales (STAI-T and STAI-S). The 20 item trait scale assesses a person’s general anxiety level (trait anxiety questionnaire) on a 4-point Likert scale and is scored from 0 to 80. The current anxiety level (state anxiety questionnaire), also includes 20 items and scored is from 0 to 80 [49]. In our study, the internal reliability of the questionnaire was found to be excellent for both the STAI-T (Cronbach’s alpha = 0.89) and STAI-S (Cronbach’s alpha = 0.91) scales.

#### Beck Depression Inventory-short form

The Beck Depression Inventory 9-item shortened version (BDI-9) was used to measure depression levels. The questionnaire measures the presence of certain symptoms of depression over the past month, such as social withdrawal, indecision, fatigue, sleep disturbance, inability to work, pessimism, lack of pleasure and satisfaction, and self-blame. It uses a 4-point Likert scale ranging from 0 to 27 points [3]. The internal reliability of the instrument was adequate in our study (Cronbach’s alpha = 0.79).

#### Perceived Stress Scale

A shortened 10-item version of the Perceived Stress Scale (PSS-10) was used to measure stress levels and subjective stress perception. The respondent is asked to rate on a five-point Likert scale how often in the past month he or she has experienced a particular feeling or thought that characterizes stress [14] the questionnaire scores from 0 to 40. The reliability of the questionnaire in our study sample was also found to be adequate (Cronbach’s alpha = 0.75).

#### EQ-5D-3L

The overall quality of life was measured by calculating the EQ-5D index of the European Quality of Life questionnaire. The questionnaire assesses health-related quality of life along five dimensions: mobility, self-care, ability to carry out usual activities of daily living, pain/discomfort, and anxiety/depression. Each dimension is assessed by a single question and is answered on a three-point scale [7, 54]. The internal reliability of the instrument was also found to be sufficient in our study (Cronbach’s alpha = 0.72).

#### Brief Illness Perception Questionnaire

We explored attitudes to illness using the abbreviated Perception of Illness Questionnaire. The questionnaire distinguishes eight dimensions of illness perception: consequences, timeline, personal control, treatment control, identity, concern, comprehensibility, and representation of emotions. Responses are given on a 10-point Likert scale, with the score ranging from 0 to 80 points. The response score calculates a total score, which represents the perceived threat associated with the disease; the higher the score, the more negative the disease image [6]. The internal consistency of the measure in our study sample was slightly below the minimum expected value of 0.7 (Cronbach’s α = 0.66), but it does indicate acceptable internal reliability for attitude scales.

### Analysis of the Emotional Graph of Illness Trajectory: Content analysis of qualitative data

During the Emotional Graph of Illness Trajectory test session the examiner recorded the following on the marking sheet: the dominant emotion of the illness trajectory and its justification, the main events of the illness process named by the patient, their content, their description, and finally the answers to the questions asked during the post-test. This data was subjected to thematic content analysis involving two independent coders. Our research team considered the aspects of Frank’s concept of illness narratives [19, 20] as characteristic of illness narratives and used them as the criteria for content analysis, thus, the theoretical basis for the categorisation that helped to form the experimental groups also provided the standard for coding. Furthermore, relying on the analytical method of [52, 42] Interpretation Theory was helpful in the analysis process. Coding was performed by the two independent coders as part of the content analysis. We provided them with the literature on which our research was based, including a detailed description and relevant sources that commented on Frank’s theory of disease narrative. Several consultations were held with independent coders to establish the appropriate theoretical knowledge, and the authors were satisfied that the coders were appropriately knowledgable.

The primary aim of the verbal content analysis was to determine the patient’s dominant narrative of the illness currently at the forefront of the narrative. During the thematic content analysis, the coders assessed the content of the marking sheets in three steps. They marked the closest illness narrative type regarding content and narrative (chaos story, restitution story or quest story), as follows. (1) the emotion associated with the disease process and the reasons for the emotion; (2) the emotional content of experiences related to events in the disease process; and (3) the content of the answers to the post-test questions was assessed according to the Frank concepts. As a result, the subject’s illness narrative was the narrative type that appeared most often in the verbal content.

### Statistical analysis

Statistical analyses were performed using SPSS version 22.0 [27]. G-Power 3.1 was used to calculate effect size values [16]. Figures were prepared using R Studio [44]. The limit of statistical significance was set at *p* < 0.05. Before analysing the data, the Kolmogorov-Smirnov test was used for normality tests on the mean values of emotion intensity, obtained using the graphical technique, and on the results obtained from the questionnaires, both for the whole study sample and for our subgroups. The Shapiro-Wilk test was used for groups with fewer than 30 members. Pearson correlation tests and Spearman rank correlation tests without a normal distribution were used to analysing the strength and direction of the correlations between the psychological factors examined. For comparisons of several independent groups, after performing Levene’s test, one-way ANOVA and Kruskal-Wallis H test were used, and Welch’s test was applied for inequality of variances. An independent samples t-test and Mann-Whitney U test were used when comparing two independent groups. Post hoc pairwise comparisons of groups were performed using Hochberg’s GT2 test for parametric tests. Mann-Whitney U test and Bonferroni test were used for non-parametric tests. Correlations between the groups were tested using the Chi-square test for variables at nominal or ordinal measurement levels.

## Results

### Descriptive statistics of the study sample

The descriptive statistics of the results obtained from the questionnaires and the graphical elicitation technique are reported for the total study sample (*n* = 140) in Table 2.

**Table 2:** Descriptive statistics of the results obtained for the total study sample. Mean scores and standard deviations (SD) of the results obtained by the use of the psychological measurement tools

Psychological variables	Mean	Standard deviation (SD)
**Emotional intensity** **(Emotional Graph of Illness Trajectory)**	59.26	20.90
**Depression (BDI-9 scale)**	5.74	4.30
**State anxiety (STAI-S scale)**	44.32	11.27
**Trait anxiety (STAI-T scale)**	45.02	10.51
**Perceived stress (PSS-10 scale)**	19.00	6.30
**Illness perception (BIPQ scale)**	38.50	13.68
**Quality of life (EQ-5D index)**	0.67	0.34

*Note*: *BDI-9 *Beck Depression Inventory-Short Form, *STAI-S *State-Trait Anxiety Inventory – State anxiety questionnaire, *STAI-T* State-Trait Anxiety Inventory – Trait anxiety questionnaire, *PSS-10* shortened version of the Perceived Stress Scale, *BIOQ* Brief Illness Perception Questionnaire, *EQ-5D* European Quality of Life questionnaire

### Examination of illness narrative types and their relation with psychological variables

Through analysis of the verbal content recorded using the graphic elicitation technique, we identified the illness narratives defined by A. W. Frank and classified each subject into the corresponding narrative type group [19, 20]. Two independent coders performed the grouping of narratives, and the results of these codings showed a discrepancy according to the results of the Chi-square test (χ²(4) = 235.369; *p* < 0.001), but it is important to note that this discrepancy appeared in 5% of the cases (7 subjects). In cases where the type of illness narrative identified by the two independent coders differed, the coding of the subject’s narrative was determined based on the involvement and assessment of a third independent coder. The illness narratives of the overall study sample were distributed as follows: 60.7% restitution story (85 individuals), 24.3% chaos story (34 individuals), and 15% quest story (21 individuals). The association of illness narrative type with sociodemographic variables was examined using one-way ANOVA and Chi-square test, with sociodemographic variables as independent variables and narrative type as a dependent variable. The findings of our ANOVA analysis showed that there was no difference between the three disease-narrative type groups in terms of age (*F*(2,137) = 0.498, *p* = 0.609) or in terms of the Chi-square test for nominal and ordinal level sociodemographic variables (sex: χ²=1.938, *p* = 0.379; area of residence: χ²=1.224, *p* = 0.874; educational qualifications: χ²=9.996, *p* = 0.616; marital status: χ²=16.382, *p* = 0.282; activity: χ²=6.8, *p* = 0.871). The type of narrative did not show a significant correlation with any sociodemographic variable.

In our hypothesis testing, we used one-way ANOVA to compare the scores of the three narrative-type groups for normally distributed variables and Kruskal-Wallis H test to compare the scores of the BDI, BIPQ, and EQ-5D index scales for non-normal distribution. In our comparison of the groups, equality of variances was met for all dependent variables (STAI-S: *F*(2,137) = 0.906, *p* = 0.406; STAI-T: *F*(2,137) = 2.292, *p* = 0.105; BDI: *F*(2,137) = 0.442, *p* = 0.644; BIPQ: *F*(2,137) = 0.044, *p* = 0.957; EQ-5D index: *F*(2,137) = 0.026, *p* = 0.974; PSS: *F*(2,137) = 0.446, *p* = 0.641; Emotional Graph of Illness Trajectory: *F*(2,137) = 1.138, *p* = 0.324).

The testing of our three hypotheses was shown to be feasible by performing the same statistical test for each group, thus the description of the hypothesis testing results refers to the same results: they are presented once for each of the disease narrative groups, and a complex interpretation can be achieved by considering the results together. Our hypothesis were that (1) patients who use chaos narrative would be characterized by the most negative psychological status and that they would be the most likely to show a great psychological burden as a result of their illness. We further hypothesized that (2) patients using the restitution narrative would be characterized by a moderate psychological state compared to the other two narrative types, with these patients showing less psychological distress than those using the chaos narrative. Finally, we hypothesize that (3) patients using the quest narrative would be characterised by the most positive psychological state, with the least psychological burden caused by their disease compared to the other two narrative types.

Our analysis showed a small effect, significant difference in the mean value intensity of the graphically depicted emotion when comparing the three narrative types (*F*(2, 137) = 3.472, MSE = 422.168, *p* = 0.034, η^2^ = 0.05). Furthermore, perceived stress (PSS) (*F*(2, 137) = 7.471, MSE = 38.362, *p* = 0.001, η^2^ = 0.1), state anxiety (STAI-S) (*F*(2, 137) = 4.124, MSE = 121.538, *p* = 0.018, η^2^ = 0.06), trait anxiety (STAI-T) (*F*(2, 137) = 5.796, MSE = 103.403, *p* = 0.004, η^2^ = 0.08), depression (BDI) (*H*(2) = 7.946, *p* = 0.019, η^2^ = 0.05), and perception of illness (BIPQ) (*H*(2) = 12.577, *p* = 0.002, η^2^ = 0.08) also showed significant differences with small and medium effects between the three groups of narrative types. (Table 3). Thus, our results show that the type of illness narrative has a small effect size, with a significant impact on the mean intensity of the emotion depicted in the Emotional Graph of Illness Trajectory and on the depression score, and a medium effect size, with a significant impact on the levels of trait anxiety, state anxiety, illness perception, and perceived stress.

According to the results of the pairwise comparison of the groups, for all the dependent variables that showed significant differences, there was no significant difference among the three groups in the mean value of the graphically depicted intensity of the emotion. However, Hochberg’s GT2 test results showed a significant difference in perceived stress between the chaos story and restitution story groups (*p* = 0.013) and between the chaos story and quest story groups (*p* = 0.001). There was also a significant difference in state anxiety between the chaos story and quest story groups (*p* = 0.022), in trait anxiety between the chaos story and quest story groups (*p* = 0.005), and between the chaos story and restitution story groups (*p* = 0.03) (Table 3). Post hoc Mann-Whitney U tests showed a significant difference in the depression scores of the chaos story and restitution story groups (*p* = 0.01) and of the chaos story and quest story groups (*p* = 0.018). Furthermore, for illness perception, there was a significant difference between the chaos story and the restitution story (*p* = 0.045), between the restitution story and the quest story groups (*p* = 0.011), and between the chaos story and the quest story (*p* = 0.001) (Table 3). Thus, our results show that the type of illness narrative had a significant effect on the average intensity of the emotion depicted in the Emotional Graph of Illness Trajectory and the trait anxiety, state anxiety, depression, perceived stress and illness perception levels. Based on the analysis of our results, our first hypothesis was supported: the chaos story was characterised by the most negative perception of illness, with the highest levels of depression, state anxiety, trait anxiety and perceived stress. As regards quality of life, our first hypothesis was not confirmed. Our second hypothesis was also supported: the restitution narrative group had a moderate psychological state compared to the other two narrative groups and had more positive depression, trait anxiety, perception of illness and perceived stress scores than the chaos narrative group. In addition, the restitution story showed the lowest emotional intensity of the disease process. Our second hypothesis regarding state anxiety and quality of life was not confirmed. Our analysis also partially supported our third hypothesis: the quest story was characterised by the most positive perception of illness and the lowest perceived stress, depression, trait anxiety and state anxiety levels, which were associated with the highest emotion intensity values for the illness process. As regards quality of life, our third hypothesis was also not confirmed (Table 3).

We explored which emotions within each narrative type showed the strongest emotional intensity as expressed by the patients and found that for patients narrating the chaos narrative, although only for one person, the despair emotion showed the strongest average emotional intensity (100%), followed by feelings of uncertainty (average intensity: 72.4%; range: 50–85%) and fear (average intensity 71.0%; range: 25–92%). In the restitution narratives, several emotions were expressed with an average intensity of 100%; security, determination, and acceptance; but each of these emotions was expressed by only one person, with fear being the other most intensely expressed emotion (average intensity: 60.86%; range: 18.75–100%). In the quest narratives, hopefulness was the most intensely expressed emotion (mean intensity: 90.5%; range: 81–100%), along with confidence (mean intensity: 95%) and trust (mean intensity 93%), but the latter two emotions appeared in only one patient’s narrative.

Our study used a Chi-square test to assess whether there is a difference in the prevalence of different narrative types between groups of patients with chronic disease and patients operated on for cancer. Our results showed no significant difference between the two groups of patients (χ²(2) = 5.491; *p* = 0.064).

**Table 3:** Mean scores and standard deviations (SD) of the emotional graph of illness trajectory and the questionnaires used, presented by illness narrative group

	Chaos story^a^	Restitution story^b^	Quest story^c^	F	H	*p*	η2	*P* value of signifficant post hoc test
**Intensity value of emotion on the Emotional Graph of Illness Trajectory**	64.27 (19.14)	55.6 (20.34)	65.96 (23.44)	3.472	-	0.034	0.05	-
**BDI**	7.41 (4.22)^b, c^	5.34 (4.41)^a^	4.67 (3.26)^a^	-	7.946	0.019	0.05	^a, b^0.01 ^a, c^0.018
**STAI-T**	49.65 (9.14)^b, c^	44.28 (11.06)^a^	40.52 (7.53)^a^	5.796	-	0.004	0.08	^a, b^0.03 ^a, c^0.005
**STAI-S**	48.59 (12.08)^c^	43.62 (11.07)	40.29 (8.80)^a^	4.124	-	0.018	0.06	^a, c^0.022
**BIPQ**	43.50 (13.44)^b, c^	38.42 (13.28)^a, c^	30.70 (12.46)^a, b^	-	12.577	0.002	0.08	^a, b^0.045 ^b, c^0.011 ^a, c^0.001
**PSS**	22.08 (5.20)^b, c^	18.54 (6.30)^a^	15.90 (6.17)^a^	7.471	-	0.001	0.10	^a, b^0.013 ^a, c^0.001
**EQ-5D**	0.6 (0.33)	0.7 (0.34)	0.67 (0.33)	-	5.516	0.06	-	-

*Note*: *BDI-9* Beck Depression Inventory-Short Form, *STAI-S* State-Trait Anxiety Inventory – State anxiety questionnaire, *STAI-T* State-Trait Anxiety Inventory – Trait anxiety questionnaire, *PSS-10* shortened version of the Perceived Stress Scale, *BIOQ* Brief Illness Perception Questionnaire, *EQ-5D* European Quality of Life questionnaire; mean scores marked with „a”, „b” and „c” indicate scores with significant difference in post hoc test

### Examining the structure of illness perception across illness narrative types

According to the hypothesis testing in the context of the investigation of the structure of illness perception across illness narrative types, our results showed that in addition to the significant difference in the overall illness perception scores described above, significant differences were found showing medium effect in the three dimensions of illness perception: treatment control (*H*(2) = 7.479, *p* = 0.024, η^2^ = 0.06), illness-related concern (*H*(2) = 10.116, *p* = 0.006, η^2^ = 0.08) and emotional representation (*H*(2) = 13.182, *p* = 0.001, η^2^ = 0.1). Thus, our results show that the type of illness narrative has a medium effect size, with a significant impact on the treatment control, illness-related concern, and emotional representation dimensions of illness perception. According to the results of post hoc Mann-Whitney U tests, the chaos story scored significantly higher on overall perception of illness (*p* = 0.045) and on its scores for the treatment control dimension (*p* = 0.049) than did the restitution story.It also scored significantly higher, in other words, more negative scores compared to the quest story for the overall perception of illness (*p* = 0.001) and for the treatment control dimension (*p* = 0.009), as well as for the illness-related concern (*p* = 0.001) and emotional representation dimensions (*p* < 0.001) (Table 4).

Furthermore, comparisons of the restitution story and quest story groups were also performed using Mann-Whitney U post hoc tests, which showed that the restitution story group had a significantly higher overall illness perception score (*p* = 0.011) and significantly higher scores for the illness-related concern (*p* = 0.012) and emotional representation dimensions (*p* = 0.006) than were found for the quest story group (Table 4). Based on the results of hypothesis testing, each of our hypotheses was supported for the illness-related concern, treatment control, and emotional representation of illness perception dimensions. Our hypotheses were not confirmed for the other disease perception dimensions,.

**Table 4:** Mean scores and standard deviations (SD) of the three groups of illness narrative types for the BIPQ scale total and its dimensions

	Chaos story^a^	Restitution story^b^	Quest story^c^	H	*p*	η2	*P* value of signifficant post hoc test
**Total illness perception score**	43.5 (13.44)^b, c^	38.42 (13.28)^a, c^	30.7 (12.46)^b, a^	12.577	0.002	0.08	a, b = 0.045 b, c = 0.011 a, c = 0.001
**Consequences dimension score**	7.73 (2.36)	7.35 (2.49)	6.09 (2.84)	5.107	0.078	-	-
**Timeline dimension score**	6.17 (2.92)	5.24 (3.29)	4.82 (3.26)	3.254	0.197	-	-
**Personal control dimension score**	5.2 (2.9)	4.21 (2.74)	3.71 (2.92)	4.223	0.121	-	-
**Treatment control dimension score**	2.82 (2.79)^b, c^	1.80 (2.08)^a^	1.19 (1.90)^a^	7.479	0.024	0.006	a, b = 0.049 a, c = 0.009
**Identity dimension score**	7.12 (12.83)	4.8 (3.0)	4.04 (2.92)	1.552	0.46	-	-
**Concern dimension score**	7.17 (2.55)^c^	6.38 (3.32)^c^	4.38 (2.71)^a, b^	10.116	0.006	0.08	a, c = 0.001 b, c = 0.012
**Comprehensibility dimension score**	2.44 (2.52)	2.21 (2.65)	2.95 (3.14)	1.558	0.459	-	-
**emotional Representation dimension score**	7.26 (2.48)^c^	6.36 (3.21)^c^	4.19 (2.82)^a, b^	13.182	0.001	0.10	a, c = < 0.001 bc = 0.006

*Note:* *BDI-9* Beck Depression Inventory-Short Form, *STAI-S* State-Trait Anxiety Inventory – State anxiety questionnaire, *STAI-T* State-Trait Anxiety Inventory – Trait anxiety questionnaire, *PSS-10* shortened version of the Perceived Stress Scale, *BIOQ* Brief Illness Perception Questionnaire, *EQ-5D* European Quality of Life questionnaire; mean scores marked with „a”, „b” and „c” indicate scores with significant difference in post hoc test

**Fig. 2:**
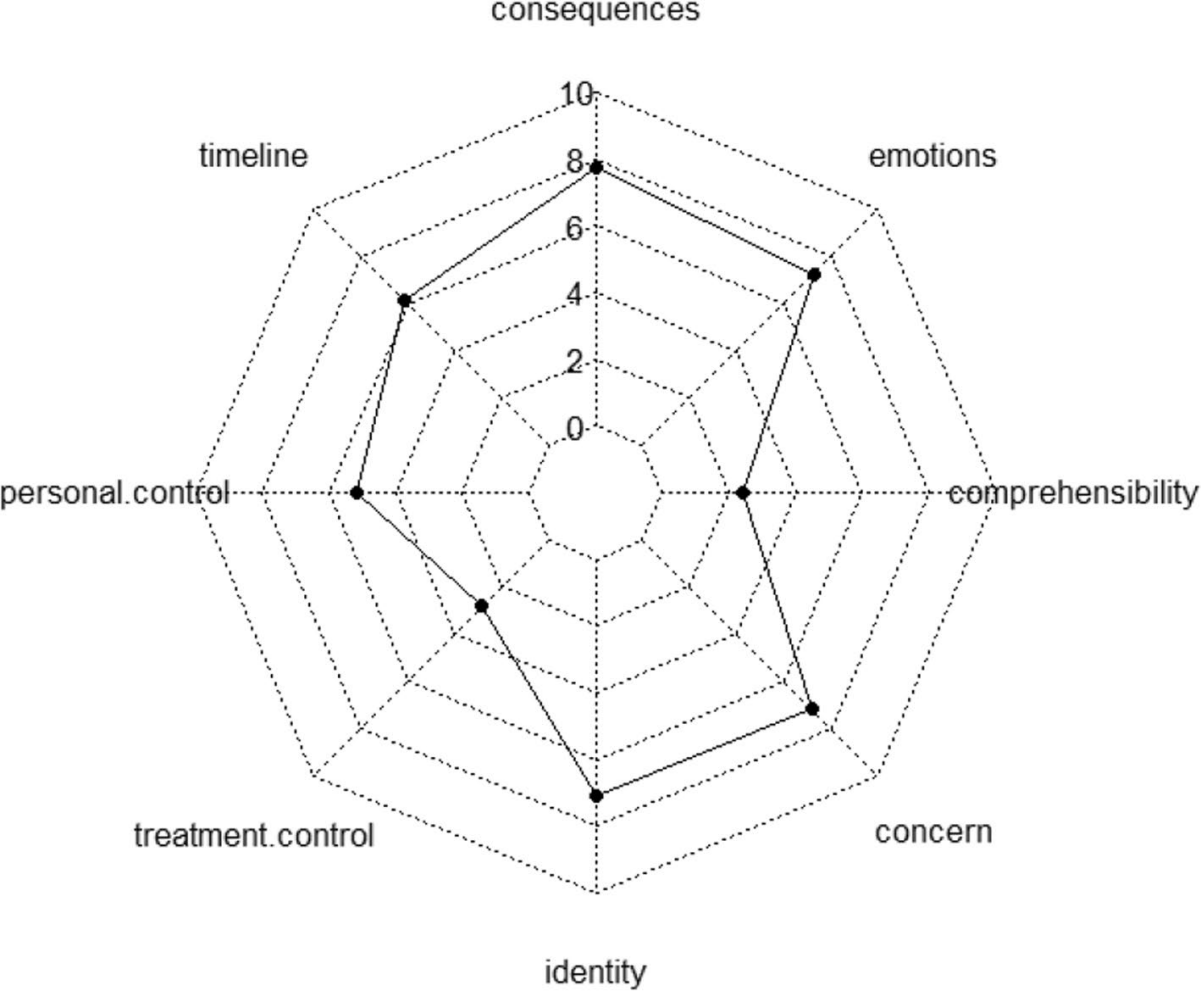
Structure of the chaos story group's perception of illness according to the scores of the eight dimensions

To summarise the characteristics of disease perception for the three illness narrative types explored in our study, the group narrating the chaos story showed the most negative perception of the disease, hence, they had the highest level of perceived threat from disease. Furthermore, the chaos story had the highest scores for the disease perception dimensions that showed significant differences, such as treatment control, concern, and emotional representation (Table 4) (Fig. 2).

**Fig. 3:**
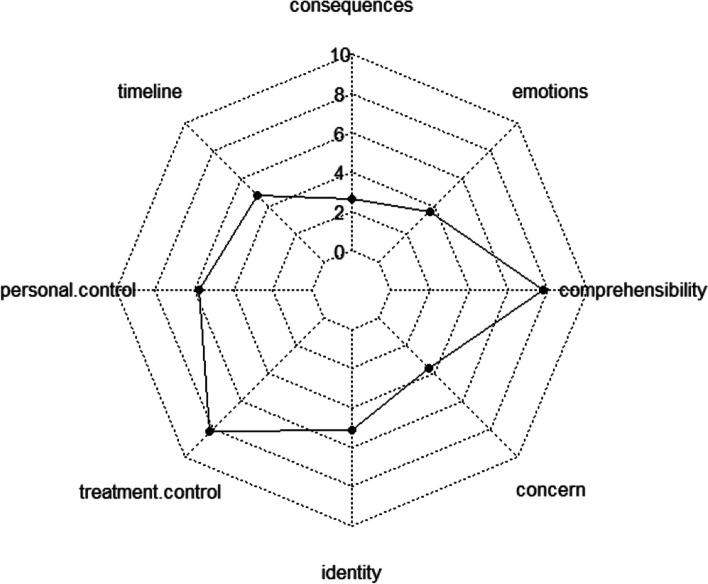
Structure of illness perception of the restitution story group according to the scores of the eight dimensions

The restitution story group is a narrative type with so-called “intermediate” disease perception characteristics, as shown by its significantly higher overall score than that found for the chaos story but a significantly lower score than that of the quest story; therefore, it shows a more positive perception of illness and a lower level of a perceived threat than the chaos story but by a more negative perception of illness and a higher level of a perceived threat than the quest story (Table 4) (Fig. 3).

**Fig. 4:**
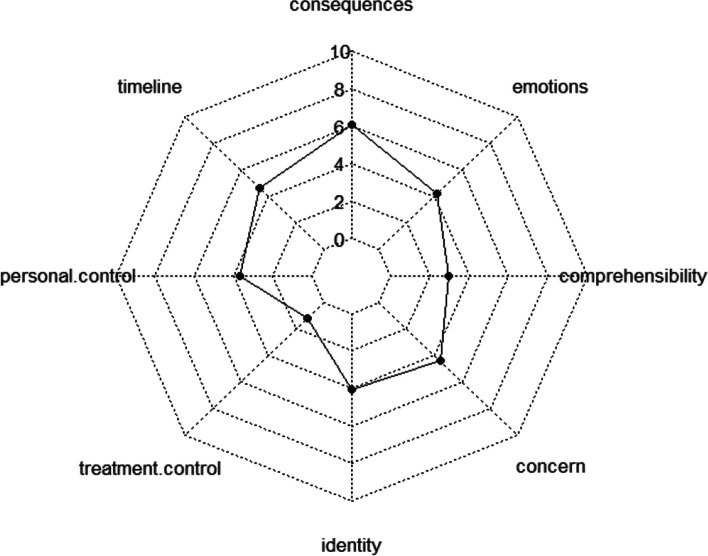
Structure of illness perception of the quest story group according to the scores of the eight dimensions

The individuals who narrated the quest story could be considered to have the most positive perception of illness, in other words, the group with the lowest perceived threat of illness. This group had the lowest scores on all dimensions when compared to the other two groups, except for the dimension of coherence (which asks about the patient’s understanding of their illness), and significantly lower scores for illness-related concern and emotional representation when compared to the groups narrating the chaos and restitution narratives, according to the results of the post hoc tests, (Table 4) (Fig. 4).

## Discussion

Our study employed a mixed quantitative and qualitative methodology to investigate the associations between illness narrative type and postoperative emotional and mood state, perceived stress, illness perception, and quality of life. We applied, at the same time, a graphical elicitation technique to map the emotional aspects of the disease narratives during the preoperative period of 140 patients with chronic disease or malignany who underwent surgery and quantitative questionnaires to assess their postoperative emotional and mood states, perceived stress level, illness perception, and quality of life.

Our results showed significant differences among the three types of illness narratives in the mean values of the intensity of the graphically depicted emotion, trait anxiety, state anxiety, depression, perceived stress and illness perception. Based on the results, our first hypothesis, that patients who narrate the chaos narrative would be characterized by the most negative psychological status and that they would be the most likely to show psychological burden as a result of the illness, was partially confirmed: our results highlight that individuals who narrated the chaos story had significantly more negative perceptions of illness and higher levels of recent depression, anxiety, and perceived stress than those who narrated the quest story. They also had significantly higher levels of anxiety, depression and perceived stress and more negative illness perception than those who narrated the restitution story, which confirmed our preliminary assumptions. Our second hypothesis, namely that the restitution narrative would be characterized by a more moderate psychological state than the other two narrative types, with these patients showing less psychological distress than the chaos narrative, was partially supported: for each of the psychological factors examined, there was an intermediate score for those narrating the restitutiton narrative and significantly lower scores for depression, trait anxiety, perceived stress, and illness perception than were found for those narrating the chaos narrative. Our third hypothesis, according to which the quest narrative would be characterised by the most positive psychological state and have the least psychological burden caused by the disease when compared to the other two narrative types was also partially supported: the patients presenting the quest story had the most positive image of their illness and more confidence in medical treatments. Their illness perception was more controllable, they felt fewer symptoms and less concern about their illness, and they were less emotionally distressed than the chaos or restitution narrative groups. We also found that the highest average value of the intensity of the graphically depicted emotion appeared among those narrating the quest story. In contrast, the lowest average value was found for the restitution story. Summarizing our results from the psychological state questionnaires, which confirmed our hypothesis, we can see that for all factors, patients reporting the chaos narrative scored the highest, followed by patients reporting the restitutiton narrative, with the patients reporting the quest story scoring the lowest, meaning that storytellers using the chaos narrative had the most negative psychological state, storytellers of the restitution narrative had a moderate psychological state, while the most positive psychological state was found for storytellers of the quest narrative. At the same time, we can see that the intensity value of the graphically plotted emotion change is highest for the quest story, almost the same, only 1.73% lower, for patients narrating the chaos story, and almost 10% lower for patients narrating the restitutiton narrative. These results are presumably due to the more effective emotion regulation processes associated with quest narratives, as opposed to the two other types of narratives. When using these effective emotion regulation strategies, the patient experiences the emotional impact of the event and change related to the disease, as indicated by the emotion intensity scores, however, at the same time as this experience, adaptive emotion regulation strategies, such as cognitive reframing or interpreting illness as a challenge, can be assumed to be activated, which may lead to a stabilisation of psychological well-being and a positive change in its direction, changes that were also seen in several dimensions of postoperative psychological state and perception of illness in relation to the quest story in another report [28].

Our results of the analysis of disease perception across the three illness narratives found three dimensions of difference. For the treatment control dimension, narrators of the chaos narrative had the highest scores, significantly different than narrators of the restitutiton and quest narratives; narrators of the restitution narrative had an intermediate value, significantly different than narrators of the quest story; and those who reported the quest narrative had the lowest scores, which means that patients who report the latter narrative type perceive their disease treatment to be most effective. In addition, there were significant differences among the narrative groups for the dimensions of illness-related concerns and emotional representation expressing the emotional impact of illness, but no significant differences were found between the chaos and restitution narrative types for these dimensions: only the quest story group showed significant differences with the chaos and restitutiton narrative groups. Our results for the disease perception dimensions of treatment control may be explained by the level of trust and commitment to the success of recovery that is characteristic of the narrative types: the most negative picture is seen in the case of the chaos narrative, the restitutiton story presents illness as a recoverable condition, and the quest story presents illness as a challenge to be overcome [57, 19]. For the concern and emotional representation dimensions, as with the factors that explain the postoperative psychological states described above, it is suspected that the more adaptive emotion regulation processes characteristic of the quest narrative may explain the significant differences that emerged when compared to the chaos and restitution narratives, while the emotion regulation strategies of patients who narrate the latter two narrative types are presumably not significantly different [28]. Our findings that there were significant differences between narrative types for the treatment control, concern, and emotional representation of illness perception dimensions while no significant differences were found for the other dimensions of illness perception, is presumably because the dimensions with significant differences may be most related to emotion regulation processes, and these dimensions may express emotional involvement more significantly, with the other dimensions perhabs less [19, 28].

Our findings for the three types of illness narratives may be due to the appearance of the quest story as a mental process that involves psychological development related to adaptive emotion regulation and the restitution story as the patient’s passive role in recovery [19, 20, 51]. In the chaos story, suffering is untold, the patient cannot find his old self nor can he build a new one, which is reflected in high levels of depression, anxiety and perceived stress, as well as in a more negative perception of illness [19, 20, 36, 51].

Our findings resonated with other research showing that the emotional aspect of the illness narrative is positively related to the emotional and mood states of the narrator [5, 41]. Results from a study of patients with breast cancer also showed that the representation of the illness during the disease process was strongly related to the degree of psychological distress [34]. Our findings for illness perception were also consistent with previous research findings, suggesting that adverse changes in illness perceptions among patients with chronic illness are associated with increased negative emotions and negative changes in perceived consequences and perceived control over the illness, which are consistent with the characteristics of illness perceptions of the group of individuals who narrated the chaos story [4]. The results of a study of patients with amyotrophic lateral sclerosis also showed a correlation between patients’ representations of their disease and their mood and that psychological factors also significantly determine the adaptive or maladaptive nature of patients’ adaptation to their disease [35]. However, in addition to the consistency with previous literature, it is important to highlight the novelty of our findings for a sample of chronic and cancer patients recovering from surgery: patients who reported a chaos narrative had the highest levels of psychological distress, patients narrating the restitution narrative had a moderate psychological state compared to the other two narrative types, and patients narrating a quest narrative can be considered to be in the most positive postoperative psychological state.

Consistent with the results of previous research, our findings showed that the restitution story was the most commonly reported in our sample of patients with chronic disease and cancer who are recovering from surgery, which differs from the study samples of previous research. According to Frank’s theory, the restitution story is the most common narrative type [19]. Thomas-MacLean [51], in a study of patients diagnosed with breast cancer, also found that patients most often told their story of illness in the form of a restitution story. This phenomenon can be explained by the technological achievements in modern Western medicine and the messages of consumer culture and the media, which view the human body as an object [17, 53]. This process can be problematic psychologically, as it can create the illusion that we can quickly ‘rebuild’ ourselves or ‘replace’ sick body parts. The damaged, diseased ‘subjective physical body’ becomes modern Western medicine’s ‘objectifiable physical body’, allowing the patient to be seen and treated as a disease. However, we cannot process the psychological changes caused by a serious physical illness from one moment to the next. Frank emphasizes that a critical strength of the narrative types is to assist professionals in building stronger relationships with their patients [20]. The goal is not to direct patients away from some narratives and toward others; instead, it is critical to emphasize that no one knows their stories better. Through listening to their stories, healthcare providers can create a space for new stories to be told.

In our study, there were no significant differences between the three disease narratives in terms of health-related quality of life as measured by the EQ-5D index: none of our hypotheses were confirmed for this psychological factor. This result may be explained by the measurement capabilities of the questionnaire. The EQ-5D score may not show significant differences because it is not an appropriate measure of differences between narratives. The EQ-5D measures health-related quality of life and is designed to compare overall quality of life across different diseases and conditions. It is, however, a very general measure and may lack sensitivity to some of the psychological or emotional factors examined in this research. In particular, when the illness narrative is strongly related to the patient’s psychological adjustment and emotions, the EQ-5D alone may not adequately capture differences, lacking sensitivity to the psychological and emotional characteristics of the groups from which the patients studied were drawn. Future research should use specific measures that are more sensitive to these differences.

### Limitations

Limitations of our research include the appearance of different sample sizes for the narrative types, which may have limited the power of the statistical analyses conducted. However, the discrepancy in group sizes can be reconciled with the practice of daily clinical health psychology care. Only 21 patients in our study sample of 140 patients narrated the quest story over the 20-month duration of the study, a proportion that reflects the experience of health psychology practice: patients report significantly lower rates of positive emotional perspectives and successful physical and psychological adjustment to their chronic illness than the opposite, negative emotional perspectives, as a chaos or restitution story. A further limitation of our study is the heterogeneity of the study sample in terms of patient diagnosis and thus disease course. Future research should aim at homogeneity of the sample and separation of chronic disease and cancer patient groups.

### Conclusions and summary

Our results provide evidence that the illness narratives of Frank’s theoretical framework adequately reflect the patient’s emotions about the disease process and his or her activity or lack of activity in the recovery process, in other words, the patient’s psychological adjustment, which are related to the emotional and mood states and stress level and perception of the illness, which are specific to the recovery process, in this case during the recovery period after surgery.

According to a summary by [50], in expressing emotions, both verbally and in writing, we label and attribute our emotions. This psychological process can reduce the subjective intensity of the emotion experienced and help us understand and reframe the triggering stressor [50]. Our results align with previous findings, highlighting the importance of exploring emotional aspects accompanying the disease process, as the emotional perspective provides insights into the emotional side of coping style during the disease process [55].

Furthermore, it is essential to emphasise the importance of the Emotional Graph of Illness Trajectory we used in exploring and constructing an emotion-focused disease narrative. The present graphic technique, following other narrative psychological tools, can help to put the experience of illness into perspective, restructure it, re-evaluate it through remembering, and thus restore the disconnectedness of life history, a psychological process that adaptively contributes to reducing the traumatic impact of illness [11, 12, 22, 32]. When clinicians attend to which type of narrative seems more critical than others, they can hear where the patient is [20]. For the patients, change cannot be hurried: it is generally preferable to accept less change than to seek to hurry change by pressing a patient toward a “better” narrative. The quest story is not a goal toward which the patient should move, nor does the chaos story represent personal or social failure. When experience becomes an object, the patient gains some distance between what is being lived and what is being told. Only at this critical distance can possible actions be seen, thus making change imaginable. Stories of the body’s pains and the mind’s fears may have to be told repeatedly before the patient begins to sort out what can be reinterpreted and changed. Based on the results of our research, we can see that getting to know and understand the patient’s illness narrative provides insight into the process of understanding and adapting to the changes caused by the illness, at the mental, emotional and social levels. By understanding these factors, the psychologist can help patients who are telling their illness narrative to realize for themselves where they are in the psychological process of recovery and to find the factors that help them cope.

In summary, storytelling can help to restore a sense of personal control and reduce feelings of suffering, which are intrapersonal needs that are fundamentally damaged when facing a severe illness [19, 39].

## Supplementary information

Additional file 1: The additional file contains the instructions for and the grid graphic of the Emotional Graph of Illness Trajectory and the marking sheet.

## Data Availability

The datasets generated and analysed during the current study are available in the Open Science Framework repository, [URL: https://osf.io/sytf5].
